# The continuity of a new era in the AE&M

**DOI:** 10.20945/2359-3997000000586

**Published:** 2023-01-01

**Affiliations:** 1 Universidade Federal do Rio Grande do Sul Department of Internal Medicine Porto Alegre RS Brazil Editor-in-Chief, Archives of Endocrinology and Metabolism. Department of Internal Medicine, Universidade Federal do Rio Grande do Sul (UFRGS), Porto Alegre, RS, Brazil. Hospital de Clínicas de Porto Alegre, Porto Alegre, RS, Brazil; Hospital de Clínicas de Porto Alegre Porto Alegre RS Brazil

DEAR COLLEAGUES,

It was 1951, under the name of *Arquivos Brasileiros de Endocrinologia e Metabologia*, when the editors Thales Martins, from basic science, and Waldemar Berardinelli, from clinical research, created the current *Archives of Endocrinology & Metabolism*.

At that time, the Journal’s main scope was to register the researches developed at the *Instituto de Endocrinologia da Santa Casa* (Endocrinological Research Center of the National Faculty of Medicine of the University of Brazil). Notwithstanding, an exception could be made for well-written, synthetic, and well-documented articles which followed the publication’s standards. One volume per year would be published, in one or more fascicles, if enough material was submitted.

In the first available online version of the Journal (1) was published a review article on steroids and others on subjects that are still widely commented nowadays: normometabolic exophthalmos, use of androgens in short stature, and the diabetogenic potential of a substance used in the laboratory (formaldehyde). These researches were promoted by the *Conselho Nacional de Pesquisa* (National Research Council), today the *Conselho Nacional de Desenvolvimento Científico e Tecnológico* (National Council for Scientific and Technological Development – CNPq). Since then, the Journal’s affirmation has greatly depended on the efforts of its founders, becoming a regular publication in 1978, under the command of Dr. Armando de Aguiar Pupo, and with the support of an editorial board primarily composed of Brazilian endocrinologists.

When I became an endocrinologist, in 1986, the *Arquivos Brasileiros de Endocrinologia e Metabologia* was already the official body for scientific dissemination of the Brazilian Society of Endocrinology and Metabolism and the Brazilian Society of Diabetes. Manuscripts in Portuguese or English by leading researchers in the field (Bernardo Liberman, Bernardo Leo Wajchenberg, Doris Rosenthal, among many others) were published. Prof. Antônio Roberto Chacra, a great diabetologist, was the editor and director responsible for the Journal, which had the support of a strong editorial commission and was hosted at *Escola Paulista de Medicina*. On that occasion, the *Arquivos Brasileiros de Endocrinologia e Metabologia* was included in the Index Medicus Latinoamericano, Excerpta Medica, Chemical Abstracts and Biological Abstracts.

Becoming a postgraduate student right after specializing in endocrinology and metabolism made me realize the importance of producing high-quality academic work and sharing my findings with the scientific community. Both my supervisor and my mentor, Prof. Helena Schmid, and the Head of the Endocrinology Service of the Hospital de Clínicas, Jorge Luis Gross, respectively, took their PhD in Ribeirão Preto, and played a key role in transmitting this perception to me.

However, high quality research requires high commitment, from having interesting and original ideas, submitting projects for financing agencies, getting research funding, working in a team and under the guidance of those who already have experience, collecting data preferably in a team, analyzing and organizing the information gathered, and, finally, writing the manuscript. And, then, comes the challenge of finding a Journal with a suitable scope to the manuscript, a high impact factor and which is preferably read by many researchers, so that there are many citations, closing the final cycle. Upon finishing my Master Degree and PhD, I published the results in international Journals and, at some point, I have written a narrative review that addressed part of the thesis specifically related to the pathogenesis of diabetic nephropathy. In general, narrative reviews are published in response to invitations from journal editors, but I submitted the article entitled “Role of protein kinase C in the development of vascular complications of diabetes mellitus” to the *Arquivos Brasileiros de Endocrinologia e Metabologia*, a review which intended to compile the effects of hyperglycemia upon the DAG-PKC pathway, vascular dysfunction related to it, and new perspectives regarding treatment of chronic vascular complications of diabetes based on the inhibition of this pathway (2). Prof. Claudio Kater was the Editor-in-chief of the Journal, responsible for the careful work that lasted almost 10 years. I received the acceptance letter via conventional mail, as was usual at that time, and that was a great satisfaction, among many others related to future research I developed over time.

In the following years, I became a Master and then PhD’s advisor, first in the Graduate Program in Health Sciences: Cardiology, at the Institute of Cardiology of Rio Grande do Sul; and later at the Faculty of Medicine at the Universidade Federal do Rio Grande do Sul (UFRGS), as a Professor of the Graduate Programs in Medical Sciences/Endocrinology and Cardiovascular Sciences/Cardiology. Since then, I have published 23 articles at the *Arquivos Brasileiros de Endocrinologia e Metabologia* (now named *Archives of Endocrinology & Metabolism*, AE&M), which makes me extremely proud of my research work and represents the teaching and training of graduate students who integrate my research group.

There were more surprises ahead. In 2007, me and Dr. André Reis were the invited editors of the cardiovascular disease and diabetes special issue of the AE&M. In January 2019, I was very flattered to be invited by the then Editor-in-chief, Dr. Marcello Bronstein, to be an associate editor. I already had experience in reviewing articles for many national and international journals, a laborious task, but very important, since peer review is needed to qualify research and share results with seriousness. The AE&M was at the height of its internationalization process at the time. The *Arquivos Brasileiros de Endocrinologia e Metabologia* became the Archives of Endocrinology and Metabolism in 2015. The modifications proposed by our dear Marcello involved writing articles exclusively in English, and the name of the Journal gradually attracted foreign authors’ manuscripts. Nowadays, international articles represent approximately 40% of all AE&M publications.

The AE&M has always sought to offer up-to-date content, in line with science and following the evolution of medicine, but under Marcello’s direction this process skyrocketed. Following the trailblazing steps of Edna Kimura, I am only the second woman to act as Editor-in-Chief for the journal, hoping to pave the way for several others. He invited me to replace him as Editor-in-chief a few months before passing away. Here we are, after eight years under his direction, showing off an impact factor of 2.032. It is a huge mission to carry on and I am sure I must dedicate great efforts to honor his legacy. I now count on the precious help of Bruno Ferraz-de-Souza as Deputy Editor-in-chief. The team is completed by all the dedicated co-editors, the new editorial board, the zealous staff of the Brazilian Society of Endocrinology and Metabolism, and I’m confident that they are committed to this mission as I am. It was a long journey to get where we are, but it is just the beginning. We must continue the work started by Thales Martins and Waldemar Berardinelli, constantly improving the AE&M. This is our goal.
